# Robotic Adrenalectomy in a Patient With Neurofibromatosis Type 1 and Pheochromocytoma

**DOI:** 10.7759/cureus.37097

**Published:** 2023-04-04

**Authors:** Samantha Zhan-Moodie, America S Revere, Lisa R Hilton, Aaron Bolduc

**Affiliations:** 1 Minimally Invasive Surgery, Medical College of Georgia, Augusta University, Augusta, USA

**Keywords:** robotic surgery, bilateral pheochromocytoma, neurofibromatosis type 1 (nf-1), neurofibromatosis 1, adrenal pheochromocytoma

## Abstract

A 27-year-old female with a past medical history of neurofibromatosis type 1 (NF1) presented with obstructive hydrocephalus due to a thalamic tumor. The neurosurgery team attempted an operative intervention twice, but both times, the patient experienced a hypertensive emergency and unstable supraventricular tachycardia upon induction of anesthesia. After the second failed surgery, a pheochromocytoma was suspected and the workup demonstrated a left pheochromocytoma. Pheochromocytomas in patients with NF1 are known to be more dangerous and labile, requiring in-depth discussion and preparation by personnel in endocrinology, anesthesia, neurosurgery, and minimally invasive surgery. Once the patient was stable and deemed fit for surgery, a robotic adrenalectomy followed by ventriculoperitoneal shunt placement began. After induction of anesthesia, the patient went into hypertensive emergency again. However, the anesthesia team was prepared and quickly resolved this with medical therapy. Minimally invasive surgeons had the patient’s live vitals displayed on their robotic monitors to increase their awareness of patient hemodynamics. This provided live feedback on the surgeons’ effect as they removed the pheochromocytoma. Surgeons also performed vein clamping to preemptively see the effects of adrenalectomy. When vein clamping demonstrated safety to proceed, adrenalectomy was completed without complication. This case not only highlights the rare pathology of a woman with NF1 with pheochromocytoma, but it also demonstrates the importance of preparedness and communication among a multidisciplinary team in complex cases to ensure a successful outcome. Novel techniques were also used in performing a robotic-assisted adrenalectomy that can aid other adrenal surgeons.

## Introduction

Neurofibromatosis type 1 (NF1) is a genetic disorder that affects one in 3,000 people [1]. It is inherited through an autosomal dominant pattern; however, about 50% of NF1 cases are the result of a de novo neurofibromin gene variation on chromosome 17 [2]. Those with NF1 typically have six or more café-au-lait macules; inguinal and/or axillary freckling; Lisch nodules, or iris hamartomas; and benign peripheral nerve sheath tumors, called neurofibromas. There is also an increased risk for other tumors in the disorder. Notably, those with NF1 are at almost fivefold increased risk of developing low-grade astrocytomas, brainstem gliomas, and high-grade gliomas, which present with increased intracranial pressure [3,4]. In this case, we report a case of pheochromocytoma in a young woman with NF1 presenting with hydrocephalus due to a brain neoplasm.

## Case presentation

A 27-year-old female with a past medical history of neurofibromatosis type 1 (NF1) and anxiety presented to the ED with persistent headaches and vomiting for weeks. She was admitted and found to have a thalamic tumor causing obstructive hydrocephalus. The neurosurgery team decided to proceed with a ventriculostomy for treatment. However, after induction of anesthesia, the patient went into supraventricular tachycardia and ventricular tachycardia and coded on the table for 15 minutes, requiring CPR and two rounds of cardioversion. After obtaining the return of spontaneous circulation, she was admitted to the neuro ICU. Nine days following the initial failed surgery, a third ventriculostomy via frontal burr hole and stereotactic biopsy were performed without issue. She remained in the neuro ICU for six more days before discharge. Biopsy revealed a low-grade glioma consistent with diffuse astrocytoma.

**Figure 1:**
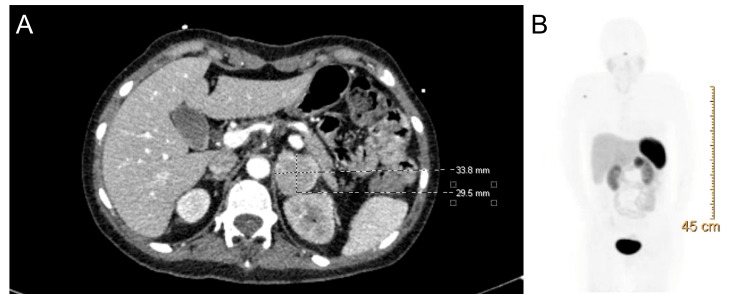
(A) CT abdomen and pelvis with IV contrast of left adrenal mass; (B) PET scan showing increased radiotracer uptake of mass superior to the left kidney. CT, computed tomography; PET, positron emission tomography; IV, intravenous

Ten days later, she returned to the ED with fatigue, headaches, and vomiting. CT head showed re-accumulation of her hydrocephalus. The neurosurgery department decided to proceed with surgical intervention again via ventriculostomy, this time with the presence of the cardiac anesthesia team. However, after induction anesthesia, the patient entered supraventricular tachycardia requiring cardioversion and hypertensive emergency with systolic blood pressure recordings in the 240s. The patient underwent one round of synchronized cardioversion and was stabilized. A right external ventricular drain via the right frontal burr hole was then placed. At this time, due to her repeated unstable hypertension and tachycardia, a pheochromocytoma was suspected and endocrinology was consulted. Additional history was obtained, and it was discovered the patient had been experiencing periods of flushing and hot spells that she referred to as *panic attacks*. Metanephrine and 24 hours of urine catecholamines were collected, which were both found to be elevated at 4,390 and 277 mcg per 24 hours, respectively. A CT abdomen and pelvis with IV contrast demonstrated a 3.9 cm × 3.4 cm × 3.7 cm left adrenal mass (Figure 1A). Precontrast, postcontrast, and arterial phase Hounsfield units were 35.80, 103.65, and 63.39, respectively. The absolute percent washout was 59.3%. PET scan showed a left adrenal gland mass with intense radiotracer uptake, consistent with pheochromocytoma (Figure 1B). 

Pheochromocytomas in patients with NF1 are known to be more dangerous and labile [5], requiring in-depth discussion and preparation for all personnel involved. Preparation began weeks before surgery. A two-part surgery was planned - first, a robotic left adrenalectomy, followed by ventriculoperitoneal shunting. The patient was put on metoprolol after her first hospitalization and then on Prazosin for 13 days before surgery to ensure appropriate beta and alpha blockade.

When the patient's vitals became more appropriate, the anesthesia team was made aware of her case and the lability of her tumor. Urology was consulted and prepared in case a partial nephrectomy was indicated. On the day of surgery, all personnel were prepared. After induction of anesthesia, the patient again went into hypertensive emergency with readings of 350/200. However, the anesthesia team was prepared and quickly resolved this with remifentanil, midazolam, fentanyl, lidocaine, propofol, nitroprusside, nitroglycerin, rocuronium, and phenylephrine.

**Figure 2:**
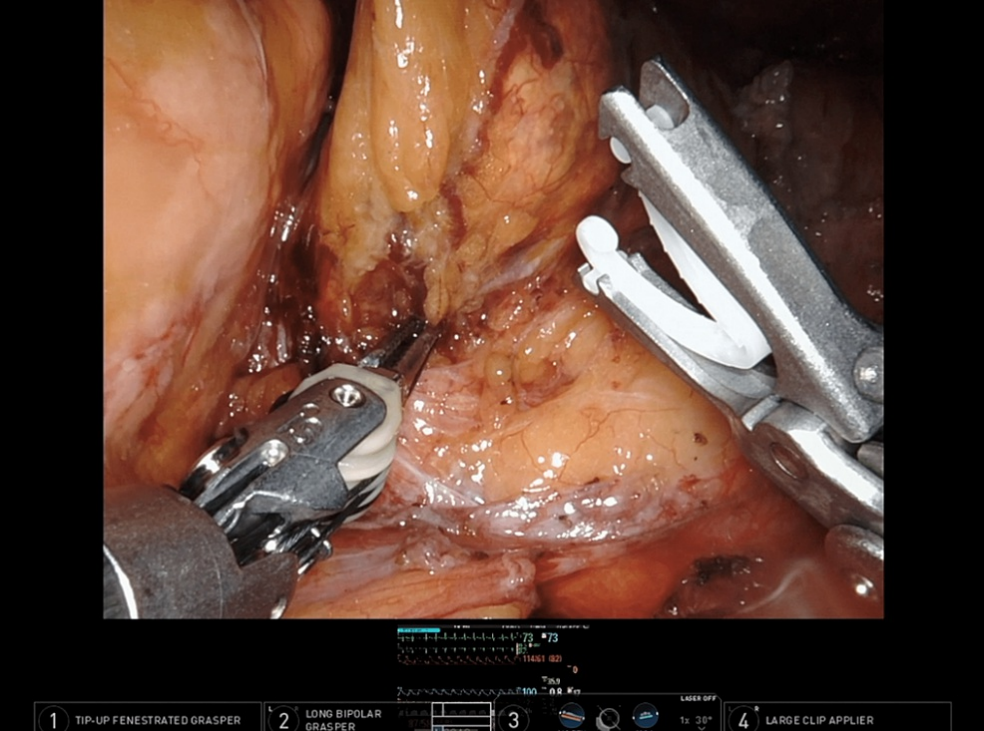
An example image of robotic monitor with the patient's live vital readings shown.

**Figure 3:**
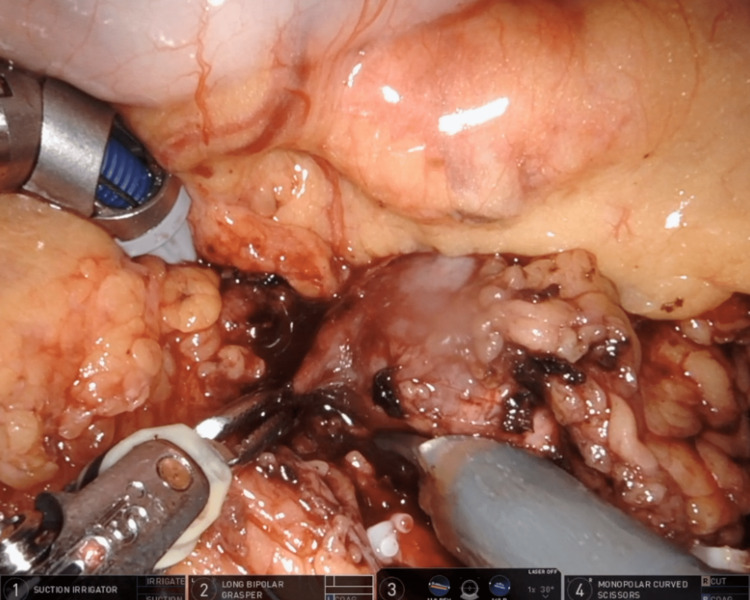
Laparoscopic view of temporary clamping of the adrenal vein in robotic adrenalectomy.

The surgery proceeded with robotic-assisted left adrenalectomy. The patient’s live vitals were displayed on the robotic monitors to increase surgeons’ awareness of patient hemodynamics (Figure 2). This provided live feedback on the surgeons’ effect on the patient as the pheochromocytoma was removed. Two adrenal veins were identified during dissection. Surgeons then performed a 1-minute clamp trial, during which the adrenal veins were temporarily clamped for 1 minute to ascertain the effects of blocking catecholamine release from the pheochromocytoma before permanently removing the tumor (Figure 3). 

When vein clamping demonstrated no hemodynamic changes and safety to proceed, the left adrenalectomy was completed without complication. The neurosurgery team was then able to place a left ventriculoperitoneal shunt without issue. Pathology reported the pheochromocytoma was removed with negative margins. At one-month postsurgery, the patient reported she was doing well with complete resolvent of headaches. Unfortunately, the patient passed away 2 years later at the age of 29 years due to her brain

cancer.

## Discussion

This is a surgically complex case that involves a rare pathology in someone with a rare disorder. Those with NF1 are at significant risk of developing brain neoplasms [3,4]. They are also at a significantly increased risk for pheochromocytomas [4,5-7]. One study reported that those with NF1 were 126 times more likely to have it than those without NF1 [5]. Pheochromocytomas typically present with hypertension, headaches, sweating, flushing, and/or heart palpitations. However, 5% to 15% present as normotensive and only 24% present with the classic triad of headaches, palpitations, and sweating [8-9], making this condition easy to miss. Particularly in those with NF1, 22% of pheochromocytomas are asymptomatic [6]. However, recent guidelines do not recommend screening asymptomatic individuals for pheochromocytoma [10]. Pheochromocytomas in patients with NF1 are known to be more dangerous and labile, and an unknown pheochromocytoma can greatly endanger a patient during surgery [11]. Physicians should have a degree of clinical suspicion and preparedness when a patient with NF1 is undergoing surgery. If a pheochromocytoma had been suspected earlier, the second episode of supraventricular tachycardia could have been avoided and reduced the number of days the patient had to be hospitalized.

Surgeons also chose to clamp the adrenal veins for 1 minute to fully assess the body’s reaction to the procedure. The patient’s live vitals were also portrayed on the surgical robot monitors to help surgeons see the patient’s hemodynamic response to their movements. These techniques are not widely discussed in current literature and could be helpful to other surgeons in similarly complex hemodynamically volatile patients. 

## Conclusions

This case not only highlights the rare pathology of a woman with NF1 with pheochromocytoma, but it also demonstrates the importance of a multidisciplinary team in complex cases to ensure a successful outcome. This case also highlights novel techniques in robotic-assisted adrenalectomy that can aid other adrenal surgeons. In patients with rare endocrine pathology, it is of utmost importance to have a knowledgeable multidisciplinary team and use novel techniques based on experience and the latest literature to ensure operative success. 
